# Ethyl 4-{[3-(adamantan-1-yl)-4-phenyl-5-sulfanyl­idene-4,5-dihydro-1*H*-1,2,4-triazol-1-yl]meth­yl}piperazine-1-carboxyl­ate

**DOI:** 10.1107/S1600536811055668

**Published:** 2012-01-31

**Authors:** Ebtehal S. Al-Abdullah, Hanadi H. Asiri, Ali A. El-Emam, Seik Weng Ng

**Affiliations:** a Department of Pharmaceutical Chemistry, College of Pharmacy, King Saud University, Riyadh 11451, Saudi Arabia; b Department of Chemistry, University of Malaya, 50603 Kuala Lumpur, Malaysia; c Chemistry Department, Faculty of Science, King Abdulaziz University, PO Box 80203 Jeddah, Saudi Arabia

## Abstract

The title mol­ecule, C_26_H_35_N_5_O_2_S, displays a chair-shaped piperazine ring, as well as a planar triazole ring whose phenyl substituent is perpendicular to the mean plane of the five-membered ring [dihedral angle = 90.00 (13)°]. The methyl­ene substituent on the piperazine ring occupies an equatorial site. Weak inter­molecular C—H⋯O hydrogen bonding is present in the crystal structure. The crystal studied was a non-merohedral twin, with a 33.9 (3)% minor component.

## Related literature

For the synthesis and application of the title compound, see: El-Emam & Ibrahim (1991 ▶). For the separation of non-morohedrally twinned diffraction data, see: Spek (2009 ▶).
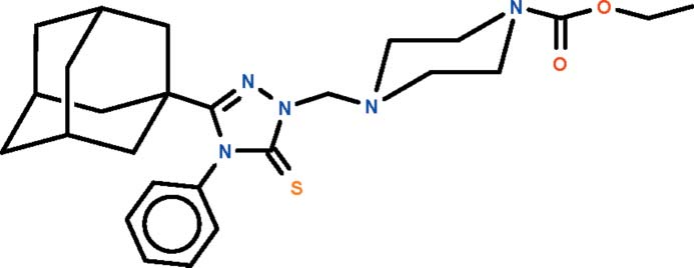


## Experimental

### 

#### Crystal data

C_26_H_35_N_5_O_2_S
*M* *_r_* = 481.65
Monoclinic, 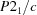

*a* = 12.0469 (6) Å
*b* = 20.9213 (10) Å
*c* = 10.3249 (5) Å
β = 109.851 (6)°
*V* = 2447.6 (2) Å^3^
*Z* = 4
Mo *K*α radiation
μ = 0.17 mm^−1^
*T* = 100 K
0.30 × 0.20 × 0.10 mm

#### Data collection

Agilent SuperNova Dual diffractometer with an Atlas detector
Absorption correction: multi-scan (*CrysAlis PRO*; Agilent, 2010 ▶) *T*_min_ = 0.952, *T*_max_ = 0.984
17138 measured reflections
5655 independent reflections
4588 reflections with *I* > 2σ(*I*)
*R*_int_ = 0.046

#### Refinement

*R*[*F* ^2^ > 2σ(*F* ^2^)] = 0.061
*wR*(*F* ^2^) = 0.156
*S* = 1.11
5655 reflections
308 parameters
H-atom parameters constrained
Δρ_max_ = 0.92 e Å^−3^
Δρ_min_ = −0.40 e Å^−3^

### 

Data collection: *CrysAlis PRO* (Agilent, 2010 ▶); cell refinement: *CrysAlis PRO*; data reduction: *CrysAlis PRO*; program(s) used to solve structure: *SHELXS97* (Sheldrick, 2008 ▶); program(s) used to refine structure: *SHELXL97* (Sheldrick, 2008 ▶); molecular graphics: *X-SEED* (Barbour, 2001 ▶); software used to prepare material for publication: *publCIF* (Westrip, 2010 ▶).

## Supplementary Material

Crystal structure: contains datablock(s) global, I. DOI: 10.1107/S1600536811055668/xu5418sup1.cif

Structure factors: contains datablock(s) I. DOI: 10.1107/S1600536811055668/xu5418Isup2.hkl

Supplementary material file. DOI: 10.1107/S1600536811055668/xu5418Isup3.cml

Additional supplementary materials:  crystallographic information; 3D view; checkCIF report

## Figures and Tables

**Table 1:** Hydrogen-bond geometry (Å, °)

*D*—H⋯*A*	*D*—H	H⋯*A*	*D*⋯*A*	*D*—H⋯*A*
C6—H6⋯O2^i^	0.95	2.48	3.406 (3)	166
C8—H8⋯O2^ii^	0.95	2.46	3.207 (3)	135

Symmetry codes: (i) 
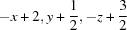
; (ii) 
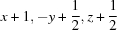
.

**Fig. 1.**
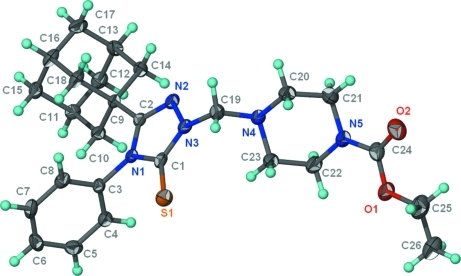
Thermal ellipsoid plot (Barbour, 2001) of C26H35N5O2S at the 70% probability level; hydrogen atoms are drawn as spheres of arbitrary radius.

## supplementary crystallographic information

### Comment

We reported the the synthesis, anti-inflammatory and analgesic properties of 3-(1-adamantyl)-4-substituted-5-mercapto-1,2,4-triazole derivatives (El-Emam & Ibrahim, 1991). The triazole ring, which possesses a secondary nitrogen site next to a double-bond sulfur, is capable of undergoing a Mannich reaction with an *N*-substituted piperazine derivative to yield a new class of chemotherapeutic compounds. The C_26_H_35_N_5_O_2_S molecule (Scheme I, Fig. 1) displays a chair-shaped piperazine ring, as well as a planar triazole ring whose phenyl substituent is perpendicular to the mean plane of the five-membered ring (dihedral ange 90.0 (1) °).

### Experimental

5-(1-Adamantyl)-4-phenyl-1,2,4-triazole-3-thiol was synthesized according to a reported procedure (El-Emam & Ibrahim, 1991). The compound (2 mmol), ethyl piperazine-1-carboxylate (2 mmol) and a 37% formaldehyde solution (0.5 ml) in ethanol (8 ml), was heated for 15 minutes. Stirring was continued for 12 h at room temperature. The product was filtered, washed with water, dried, and recrystallized from ethanol to yield (80%) of the title compound as colorless crystals, m.p. 465–467 K.

### Refinement

Carbon-bound H-atoms were placed in calculated positions [C–H 0.95 to 1.00 Å, *U*_iso_(H) 1.2 to 1.5*U*_eq_(C)] and were included in the refinement in the riding model approximation.

The crystal studied is a non-merohedral twin. The twin components were separated by using *PLATON* (Spek, 2009).

### Crystal data

**Table:**

C_26_H_35_N_5_O_2_S	*F*(000) = 1032
*M**_r_* = 481.65	*D*_x_ = 1.307 Mg m^−^^3^
Monoclinic, *P*2_1_/*c*	Mo *K*α radiation, λ = 0.71073 Å
Hall symbol: -P 2ybc	Cell parameters from 5944 reflections
*a* = 12.0469 (6) Å	θ = 2.3–27.5°
*b* = 20.9213 (10) Å	µ = 0.17 mm^−^^1^
*c* = 10.3249 (5) Å	*T* = 100 K
β = 109.851 (6)°	Prism, colorless
*V* = 2447.6 (2) Å^3^	0.30 × 0.20 × 0.10 mm
*Z* = 4	

### Data collection

**Table:**

Agilent SuperNova Dual diffractometer with an Atlas detector	5655 independent reflections
Radiation source: SuperNova (Mo) X-ray Source	4588 reflections with *I* > 2σ(*I*)
Mirror	*R*_int_ = 0.046
Detector resolution: 10.4041 pixels mm^-1^	θ_max_ = 27.6°, θ_min_ = 2.3°
ω scan	*h* = −15→15
Absorption correction: multi-scan (CrysAlis PRO; Agilent, 2010)	*k* = −27→27
*T*_min_ = 0.952, *T*_max_ = 0.984	*l* = −10→13
17138 measured reflections	

### Refinement

**Table:**

Refinement on *F*^2^	Primary atom site location: structure-invariant direct methods
Least-squares matrix: full	Secondary atom site location: difference Fourier map
*R*[*F*^2^ > 2σ(*F*^2^)] = 0.061	Hydrogen site location: inferred from neighbouring sites
*wR*(*F*^2^) = 0.156	H-atom parameters constrained
*S* = 1.11	*w* = 1/[σ^2^(*F*_o_^2^) + (0.0559*P*)^2^ + 1.7988*P*] where *P* = (*F*_o_^2^ + 2*F*_c_^2^)/3
5655 reflections	(Δ/σ)_max_ = 0.001
308 parameters	Δρ_max_ = 0.92 e Å^−^^3^
0 restraints	Δρ_min_ = −0.40 e Å^−^^3^

### Fractional atomic coordinates and isotropic or equivalent isotropic displacement parameters (Å^2^)

**Table:**

	*x*	*y*	*z*	*U*_iso_*/*U*_eq_	
S1	1.07961 (6)	0.35628 (3)	0.77520 (7)	0.02400 (17)	
N2	1.06373 (17)	0.35443 (10)	0.3905 (2)	0.0199 (4)	
N1	1.14387 (16)	0.41508 (9)	0.5741 (2)	0.0162 (4)	
N3	1.03774 (17)	0.33163 (10)	0.5027 (2)	0.0191 (4)	
N4	0.85438 (18)	0.27336 (10)	0.4214 (2)	0.0221 (5)	
N5	0.61492 (19)	0.24428 (11)	0.3815 (3)	0.0304 (5)	
O1	0.47688 (16)	0.25081 (9)	0.4818 (2)	0.0302 (4)	
O2	0.50958 (18)	0.15641 (9)	0.3949 (2)	0.0334 (5)	
C1	1.0859 (2)	0.36674 (11)	0.6181 (3)	0.0185 (5)	
C2	1.12871 (19)	0.40508 (11)	0.4359 (2)	0.0161 (5)	
C3	1.2096 (2)	0.46416 (11)	0.6668 (2)	0.0158 (5)	
C4	1.1530 (2)	0.52024 (12)	0.6799 (3)	0.0197 (5)	
H4	1.0716	0.5262	0.6288	0.024*	
C5	1.2166 (2)	0.56774 (13)	0.7686 (3)	0.0243 (6)	
H5	1.1790	0.6065	0.7784	0.029*	
C6	1.3355 (2)	0.55824 (14)	0.8428 (3)	0.0263 (6)	
H6	1.3796	0.5906	0.9029	0.032*	
C7	1.3895 (2)	0.50143 (14)	0.8290 (3)	0.0265 (6)	
H7	1.4707	0.4951	0.8803	0.032*	
C8	1.3270 (2)	0.45372 (12)	0.7418 (2)	0.0197 (5)	
H8	1.3642	0.4146	0.7337	0.024*	
C9	1.18374 (19)	0.44264 (11)	0.3488 (2)	0.0157 (5)	
C10	1.1833 (2)	0.51576 (12)	0.3709 (3)	0.0181 (5)	
H10A	1.1011	0.5309	0.3492	0.022*	
H10B	1.2273	0.5259	0.4686	0.022*	
C11	1.2406 (2)	0.55027 (12)	0.2784 (3)	0.0190 (5)	
H11	1.2398	0.5974	0.2943	0.023*	
C12	1.1715 (2)	0.53586 (12)	0.1271 (3)	0.0226 (5)	
H12A	1.2081	0.5583	0.0674	0.027*	
H12B	1.0893	0.5512	0.1035	0.027*	
C13	1.1723 (2)	0.46359 (13)	0.1033 (3)	0.0243 (6)	
H13	1.1270	0.4539	0.0045	0.029*	
C14	1.1149 (2)	0.42896 (13)	0.1953 (3)	0.0226 (5)	
H14A	1.1140	0.3824	0.1782	0.027*	
H14B	1.0322	0.4435	0.1723	0.027*	
C15	1.3685 (2)	0.52770 (12)	0.3139 (3)	0.0211 (5)	
H15A	1.4062	0.5504	0.2555	0.025*	
H15B	1.4136	0.5376	0.4114	0.025*	
C16	1.3704 (2)	0.45559 (12)	0.2898 (3)	0.0231 (5)	
H16	1.4538	0.4408	0.3122	0.028*	
C17	1.2999 (2)	0.44045 (13)	0.1382 (3)	0.0260 (6)	
H17A	1.3369	0.4620	0.0777	0.031*	
H17B	1.3008	0.3938	0.1224	0.031*	
C18	1.3131 (2)	0.42047 (12)	0.3820 (3)	0.0209 (5)	
H18A	1.3584	0.4293	0.4799	0.025*	
H18B	1.3149	0.3738	0.3669	0.025*	
C19	0.9811 (2)	0.26971 (11)	0.4941 (3)	0.0214 (5)	
H19A	0.9957	0.2531	0.5882	0.026*	
H19B	1.0165	0.2393	0.4457	0.026*	
C20	0.8097 (2)	0.20785 (13)	0.3899 (3)	0.0288 (6)	
H20A	0.8220	0.1840	0.4764	0.035*	
H20B	0.8531	0.1856	0.3372	0.035*	
C21	0.6788 (2)	0.21007 (15)	0.3057 (4)	0.0373 (7)	
H21A	0.6671	0.2318	0.2169	0.045*	
H21B	0.6476	0.1660	0.2859	0.045*	
C22	0.6601 (2)	0.30815 (13)	0.4241 (3)	0.0260 (6)	
H22A	0.6190	0.3268	0.4836	0.031*	
H22B	0.6444	0.3358	0.3420	0.031*	
C23	0.7922 (2)	0.30593 (12)	0.5025 (3)	0.0244 (6)	
H23A	0.8229	0.3500	0.5237	0.029*	
H23B	0.8070	0.2831	0.5908	0.029*	
C24	0.5325 (2)	0.21268 (12)	0.4181 (3)	0.0244 (6)	
C25	0.3970 (3)	0.21595 (15)	0.5362 (4)	0.0396 (8)	
H25A	0.4430	0.1885	0.6141	0.047*	
H25B	0.3439	0.1883	0.4637	0.047*	
C26	0.3270 (3)	0.26259 (15)	0.5833 (3)	0.0378 (7)	
H26A	0.2717	0.2398	0.6178	0.057*	
H26B	0.2829	0.2901	0.5061	0.057*	
H26C	0.3800	0.2889	0.6572	0.057*	

### Atomic displacement parameters (Å^2^)

**Table:**

	*U*^11^	*U*^22^	*U*^33^	*U*^12^	*U*^13^	*U*^23^
S1	0.0274 (3)	0.0241 (3)	0.0246 (3)	−0.0048 (2)	0.0142 (3)	0.0009 (3)
N2	0.0183 (10)	0.0185 (10)	0.0267 (11)	−0.0019 (8)	0.0124 (9)	−0.0006 (9)
N1	0.0144 (9)	0.0165 (10)	0.0198 (10)	−0.0024 (7)	0.0087 (8)	−0.0013 (8)
N3	0.0194 (10)	0.0172 (10)	0.0254 (11)	−0.0037 (8)	0.0136 (9)	−0.0013 (9)
N4	0.0216 (10)	0.0192 (10)	0.0295 (12)	−0.0051 (8)	0.0139 (9)	−0.0057 (9)
N5	0.0229 (11)	0.0304 (12)	0.0428 (14)	−0.0083 (9)	0.0178 (10)	−0.0138 (11)
O1	0.0272 (10)	0.0253 (10)	0.0445 (12)	−0.0067 (8)	0.0206 (9)	−0.0046 (9)
O2	0.0336 (11)	0.0199 (10)	0.0443 (12)	−0.0039 (8)	0.0100 (9)	−0.0032 (9)
C1	0.0135 (11)	0.0154 (11)	0.0288 (13)	0.0002 (8)	0.0101 (10)	−0.0001 (10)
C2	0.0130 (10)	0.0156 (11)	0.0208 (12)	0.0002 (8)	0.0074 (9)	−0.0009 (9)
C3	0.0151 (11)	0.0177 (11)	0.0159 (11)	−0.0037 (9)	0.0068 (9)	0.0011 (9)
C4	0.0171 (11)	0.0222 (13)	0.0204 (12)	−0.0007 (9)	0.0073 (10)	−0.0004 (10)
C5	0.0312 (14)	0.0222 (13)	0.0238 (13)	−0.0026 (10)	0.0150 (11)	−0.0035 (11)
C6	0.0275 (13)	0.0327 (15)	0.0201 (13)	−0.0164 (11)	0.0098 (11)	−0.0058 (11)
C7	0.0152 (11)	0.0454 (17)	0.0182 (12)	−0.0065 (11)	0.0049 (10)	0.0013 (12)
C8	0.0182 (11)	0.0257 (13)	0.0168 (12)	0.0018 (9)	0.0081 (10)	0.0047 (10)
C9	0.0139 (10)	0.0168 (11)	0.0179 (11)	−0.0017 (8)	0.0074 (9)	−0.0009 (9)
C10	0.0187 (11)	0.0180 (12)	0.0198 (12)	−0.0007 (9)	0.0094 (10)	−0.0009 (10)
C11	0.0218 (12)	0.0166 (11)	0.0209 (12)	−0.0002 (9)	0.0103 (10)	0.0021 (10)
C12	0.0214 (12)	0.0278 (13)	0.0200 (12)	0.0019 (10)	0.0089 (10)	0.0055 (11)
C13	0.0263 (13)	0.0317 (14)	0.0170 (12)	−0.0070 (11)	0.0100 (11)	−0.0046 (11)
C14	0.0210 (12)	0.0280 (13)	0.0214 (13)	−0.0078 (10)	0.0108 (11)	−0.0052 (11)
C15	0.0189 (11)	0.0236 (13)	0.0225 (12)	−0.0055 (9)	0.0094 (10)	0.0013 (10)
C16	0.0185 (11)	0.0245 (13)	0.0308 (14)	0.0035 (10)	0.0144 (11)	0.0066 (11)
C17	0.0328 (14)	0.0221 (13)	0.0325 (15)	−0.0021 (11)	0.0234 (12)	−0.0031 (11)
C18	0.0158 (11)	0.0223 (12)	0.0277 (13)	0.0033 (9)	0.0114 (10)	0.0068 (11)
C19	0.0221 (12)	0.0147 (12)	0.0301 (14)	−0.0041 (9)	0.0124 (11)	−0.0018 (10)
C20	0.0298 (14)	0.0217 (13)	0.0408 (16)	−0.0058 (10)	0.0199 (13)	−0.0115 (12)
C21	0.0288 (15)	0.0368 (17)	0.0490 (19)	−0.0089 (12)	0.0165 (14)	−0.0236 (15)
C22	0.0241 (13)	0.0228 (13)	0.0357 (15)	−0.0023 (10)	0.0164 (12)	−0.0019 (12)
C23	0.0241 (13)	0.0194 (13)	0.0339 (15)	−0.0052 (10)	0.0152 (12)	−0.0085 (11)
C24	0.0193 (12)	0.0219 (13)	0.0295 (14)	−0.0001 (10)	0.0051 (11)	0.0001 (11)
C25	0.0350 (16)	0.0367 (17)	0.053 (2)	−0.0081 (13)	0.0231 (15)	0.0045 (15)
C26	0.0297 (15)	0.0428 (18)	0.0458 (18)	0.0003 (13)	0.0192 (14)	0.0069 (15)

### Geometric parameters (Å, °)

**Table:**

S1—C1	1.664 (3)	C11—H11	1.0000
N2—C2	1.306 (3)	C12—C13	1.532 (4)
N2—N3	1.384 (3)	C12—H12A	0.9900
N1—C2	1.392 (3)	C12—H12B	0.9900
N1—C1	1.390 (3)	C13—C14	1.533 (3)
N1—C3	1.443 (3)	C13—C17	1.534 (4)
N3—C1	1.351 (3)	C13—H13	1.0000
N3—C19	1.453 (3)	C14—H14A	0.9900
N4—C19	1.457 (3)	C14—H14B	0.9900
N4—C23	1.467 (3)	C15—C16	1.530 (3)
N4—C20	1.468 (3)	C15—H15A	0.9900
N5—C24	1.350 (3)	C15—H15B	0.9900
N5—C22	1.453 (3)	C16—C17	1.539 (4)
N5—C21	1.457 (4)	C16—C18	1.539 (3)
O1—C24	1.348 (3)	C16—H16	1.0000
O1—C25	1.463 (3)	C17—H17A	0.9900
O2—C24	1.214 (3)	C17—H17B	0.9900
C2—C9	1.507 (3)	C18—H18A	0.9900
C3—C8	1.380 (3)	C18—H18B	0.9900
C3—C4	1.386 (3)	C19—H19A	0.9900
C4—C5	1.392 (4)	C19—H19B	0.9900
C4—H4	0.9500	C20—C21	1.522 (4)
C5—C6	1.390 (4)	C20—H20A	0.9900
C5—H5	0.9500	C20—H20B	0.9900
C6—C7	1.385 (4)	C21—H21A	0.9900
C6—H6	0.9500	C21—H21B	0.9900
C7—C8	1.383 (4)	C22—C23	1.521 (3)
C7—H7	0.9500	C22—H22A	0.9900
C8—H8	0.9500	C22—H22B	0.9900
C9—C10	1.547 (3)	C23—H23A	0.9900
C9—C14	1.546 (3)	C23—H23B	0.9900
C9—C18	1.548 (3)	C25—C26	1.476 (4)
C10—C11	1.536 (3)	C25—H25A	0.9900
C10—H10A	0.9900	C25—H25B	0.9900
C10—H10B	0.9900	C26—H26A	0.9800
C11—C12	1.530 (3)	C26—H26B	0.9800
C11—C15	1.532 (3)	C26—H26C	0.9800
			
C2—N2—N3	104.9 (2)	C9—C14—H14B	109.6
C2—N1—C1	108.66 (19)	H14A—C14—H14B	108.1
C2—N1—C3	129.67 (19)	C16—C15—C11	109.51 (19)
C1—N1—C3	121.6 (2)	C16—C15—H15A	109.8
C1—N3—N2	113.53 (19)	C11—C15—H15A	109.8
C1—N3—C19	126.2 (2)	C16—C15—H15B	109.8
N2—N3—C19	119.4 (2)	C11—C15—H15B	109.8
C19—N4—C23	112.3 (2)	H15A—C15—H15B	108.2
C19—N4—C20	107.84 (19)	C15—C16—C17	109.6 (2)
C23—N4—C20	109.9 (2)	C15—C16—C18	109.6 (2)
C24—N5—C22	126.9 (2)	C17—C16—C18	108.8 (2)
C24—N5—C21	118.8 (2)	C15—C16—H16	109.6
C22—N5—C21	113.7 (2)	C17—C16—H16	109.6
C24—O1—C25	113.1 (2)	C18—C16—H16	109.6
N3—C1—N1	102.8 (2)	C13—C17—C16	109.7 (2)
N3—C1—S1	129.78 (19)	C13—C17—H17A	109.7
N1—C1—S1	127.46 (19)	C16—C17—H17A	109.7
N2—C2—N1	110.1 (2)	C13—C17—H17B	109.7
N2—C2—C9	122.7 (2)	C16—C17—H17B	109.7
N1—C2—C9	127.1 (2)	H17A—C17—H17B	108.2
C8—C3—C4	121.6 (2)	C16—C18—C9	110.43 (19)
C8—C3—N1	119.1 (2)	C16—C18—H18A	109.6
C4—C3—N1	119.3 (2)	C9—C18—H18A	109.6
C3—C4—C5	119.3 (2)	C16—C18—H18B	109.6
C3—C4—H4	120.4	C9—C18—H18B	109.6
C5—C4—H4	120.4	H18A—C18—H18B	108.1
C6—C5—C4	119.7 (2)	N3—C19—N4	111.9 (2)
C6—C5—H5	120.2	N3—C19—H19A	109.2
C4—C5—H5	120.2	N4—C19—H19A	109.2
C7—C6—C5	119.9 (2)	N3—C19—H19B	109.2
C7—C6—H6	120.1	N4—C19—H19B	109.2
C5—C6—H6	120.1	H19A—C19—H19B	107.9
C6—C7—C8	121.0 (2)	N4—C20—C21	109.2 (2)
C6—C7—H7	119.5	N4—C20—H20A	109.8
C8—C7—H7	119.5	C21—C20—H20A	109.8
C3—C8—C7	118.6 (2)	N4—C20—H20B	109.8
C3—C8—H8	120.7	C21—C20—H20B	109.8
C7—C8—H8	120.7	H20A—C20—H20B	108.3
C2—C9—C10	113.77 (19)	N5—C21—C20	110.0 (2)
C2—C9—C14	108.89 (19)	N5—C21—H21A	109.7
C10—C9—C14	107.90 (19)	C20—C21—H21A	109.7
C2—C9—C18	108.98 (19)	N5—C21—H21B	109.7
C10—C9—C18	108.48 (19)	C20—C21—H21B	109.7
C14—C9—C18	108.7 (2)	H21A—C21—H21B	108.2
C11—C10—C9	110.37 (19)	N5—C22—C23	110.5 (2)
C11—C10—H10A	109.6	N5—C22—H22A	109.6
C9—C10—H10A	109.6	C23—C22—H22A	109.6
C11—C10—H10B	109.6	N5—C22—H22B	109.6
C9—C10—H10B	109.6	C23—C22—H22B	109.6
H10A—C10—H10B	108.1	H22A—C22—H22B	108.1
C12—C11—C15	109.8 (2)	N4—C23—C22	110.8 (2)
C12—C11—C10	109.8 (2)	N4—C23—H23A	109.5
C15—C11—C10	109.5 (2)	C22—C23—H23A	109.5
C12—C11—H11	109.2	N4—C23—H23B	109.5
C15—C11—H11	109.2	C22—C23—H23B	109.5
C10—C11—H11	109.2	H23A—C23—H23B	108.1
C11—C12—C13	109.1 (2)	O2—C24—O1	123.5 (2)
C11—C12—H12A	109.9	O2—C24—N5	124.0 (3)
C13—C12—H12A	109.9	O1—C24—N5	112.4 (2)
C11—C12—H12B	109.9	O1—C25—C26	108.7 (2)
C13—C12—H12B	109.9	O1—C25—H25A	109.9
H12A—C12—H12B	108.3	C26—C25—H25A	109.9
C14—C13—C12	109.8 (2)	O1—C25—H25B	109.9
C14—C13—C17	109.4 (2)	C26—C25—H25B	109.9
C12—C13—C17	109.5 (2)	H25A—C25—H25B	108.3
C14—C13—H13	109.4	C25—C26—H26A	109.5
C12—C13—H13	109.4	C25—C26—H26B	109.5
C17—C13—H13	109.4	H26A—C26—H26B	109.5
C13—C14—C9	110.48 (19)	C25—C26—H26C	109.5
C13—C14—H14A	109.6	H26A—C26—H26C	109.5
C9—C14—H14A	109.6	H26B—C26—H26C	109.5
C13—C14—H14B	109.6		
			
C2—N2—N3—C1	−0.4 (3)	C11—C12—C13—C14	59.8 (3)
C2—N2—N3—C19	−170.7 (2)	C11—C12—C13—C17	−60.3 (3)
N2—N3—C1—N1	1.0 (3)	C12—C13—C14—C9	−60.4 (3)
C19—N3—C1—N1	170.5 (2)	C17—C13—C14—C9	59.8 (3)
N2—N3—C1—S1	−178.88 (18)	C2—C9—C14—C13	−177.0 (2)
C19—N3—C1—S1	−9.4 (4)	C10—C9—C14—C13	59.1 (3)
C2—N1—C1—N3	−1.2 (2)	C18—C9—C14—C13	−58.4 (3)
C3—N1—C1—N3	−179.45 (19)	C12—C11—C15—C16	−60.3 (3)
C2—N1—C1—S1	178.72 (18)	C10—C11—C15—C16	60.4 (3)
C3—N1—C1—S1	0.5 (3)	C11—C15—C16—C17	59.3 (3)
N3—N2—C2—N1	−0.4 (2)	C11—C15—C16—C18	−60.1 (3)
N3—N2—C2—C9	175.8 (2)	C14—C13—C17—C16	−60.6 (3)
C1—N1—C2—N2	1.0 (3)	C12—C13—C17—C16	59.7 (3)
C3—N1—C2—N2	179.1 (2)	C15—C16—C17—C13	−59.2 (3)
C1—N1—C2—C9	−175.0 (2)	C18—C16—C17—C13	60.6 (3)
C3—N1—C2—C9	3.1 (4)	C15—C16—C18—C9	59.7 (3)
C2—N1—C3—C8	−89.4 (3)	C17—C16—C18—C9	−60.1 (3)
C1—N1—C3—C8	88.4 (3)	C2—C9—C18—C16	177.3 (2)
C2—N1—C3—C4	91.4 (3)	C10—C9—C18—C16	−58.3 (3)
C1—N1—C3—C4	−90.7 (3)	C14—C9—C18—C16	58.8 (3)
C8—C3—C4—C5	1.3 (4)	C1—N3—C19—N4	110.8 (3)
N1—C3—C4—C5	−179.6 (2)	N2—N3—C19—N4	−80.2 (3)
C3—C4—C5—C6	−0.2 (4)	C23—N4—C19—N3	−71.6 (3)
C4—C5—C6—C7	−0.6 (4)	C20—N4—C19—N3	167.1 (2)
C5—C6—C7—C8	0.2 (4)	C19—N4—C20—C21	−176.4 (2)
C4—C3—C8—C7	−1.7 (4)	C23—N4—C20—C21	60.9 (3)
N1—C3—C8—C7	179.2 (2)	C24—N5—C21—C20	−117.4 (3)
C6—C7—C8—C3	0.9 (4)	C22—N5—C21—C20	55.1 (3)
N2—C2—C9—C10	141.9 (2)	N4—C20—C21—N5	−58.0 (3)
N1—C2—C9—C10	−42.6 (3)	C24—N5—C22—C23	118.9 (3)
N2—C2—C9—C14	21.5 (3)	C21—N5—C22—C23	−52.9 (3)
N1—C2—C9—C14	−163.0 (2)	C19—N4—C23—C22	−179.4 (2)
N2—C2—C9—C18	−96.9 (3)	C20—N4—C23—C22	−59.3 (3)
N1—C2—C9—C18	78.6 (3)	N5—C22—C23—N4	54.2 (3)
C2—C9—C10—C11	−179.92 (19)	C25—O1—C24—O2	7.5 (4)
C14—C9—C10—C11	−59.0 (2)	C25—O1—C24—N5	−173.4 (2)
C18—C9—C10—C11	58.6 (2)	C22—N5—C24—O2	−169.5 (3)
C9—C10—C11—C12	60.4 (2)	C21—N5—C24—O2	1.9 (4)
C9—C10—C11—C15	−60.2 (3)	C22—N5—C24—O1	11.4 (4)
C15—C11—C12—C13	60.6 (3)	C21—N5—C24—O1	−177.2 (2)
C10—C11—C12—C13	−59.9 (3)	C24—O1—C25—C26	−171.0 (2)

### Hydrogen-bond geometry (Å, °)

**Table:**

*D*—H···*A*	*D*—H	H···*A*	*D*···*A*	*D*—H···*A*
C6—H6···O2^i^	0.95	2.48	3.406 (3)	166
C8—H8···O2^ii^	0.95	2.46	3.207 (3)	135

Symmetry codes: (i) −*x*+2, *y*+1/2, −*z*+3/2; (ii) *x*+1, −*y*+1/2, *z*+1/2.
